# Cathepsins in oral diseases: mechanisms and therapeutic implications

**DOI:** 10.3389/fimmu.2023.1203071

**Published:** 2023-06-02

**Authors:** Hao Jiang, Zuoxiang Dong, Xiaomin Xia, Xue Li

**Affiliations:** ^1^ Department of Stomatology, The Affiliated Hospital of Qingdao University, Qingdao University, Qingdao, China; ^2^ School of Stomatology, Qingdao University, Qingdao, China; ^3^ Department of Neurosurgery, The Affiliated Hospital of Qingdao University, Qingdao University, Qingdao, China

**Keywords:** cathepsin, oral diseases, inflammation, molecular mechanism, oral diseases treatment

## Abstract

Cathepsins are a type of lysosomal globulin hydrolase and are crucial for many physiological processes, including the resorption of bone matrix, innate immunity, apoptosis, proliferation, metastasis, autophagy, and angiogenesis. Findings regarding their functions in human physiological processes and disorders have drawn extensive attention. In this review, we will focus on the relationship between cathepsins and oral diseases. We highlight the structural and functional properties of cathepsins related to oral diseases, as well as the regulatory mechanisms in tissue and cells and their therapeutic uses. Elucidating the associated mechanism between cathepsins and oral diseases is thought to be a promising strategy for the treatment of oral diseases and may be a starting point for further studies at the molecular level.

## 1 Introduction

The most prevalent diseases in the world include oral diseases, which are further categorized into caries, periodontitis, periapical periodontitis, oral cancer, oral lichen planus, etc. Oral diseases are undoubtedly a more serious and widespread public health issue among socially marginalized and disadvantaged communities, but they have received less attention than other chronic diseases. Chronic progressive processes are frequently accompanied by pain and intense discomfort, which may impair one’s ability to work and learn and lower one’s quality of life (1). Simultaneously, unsightly dentition and ill-fitting dentures can affect self-esteem and exacerbate social anxiety (2). Besides, oral diseases are prone to recurrent attacks and protracted treatments, which imposes a huge economic burden on individuals and healthcare systems. The negative impacts on chewing, speech, appearance, and financial burden all push us to prioritize oral health. Nevertheless, the uncertain relevant molecular pathways of oral diseases render the current clinical oral treatment ineffective. Consequently, it’s imperative to thoroughly understand the associated mechanisms in order to achieve a better therapeutic effect for oral diseases.

**Figure 1:**
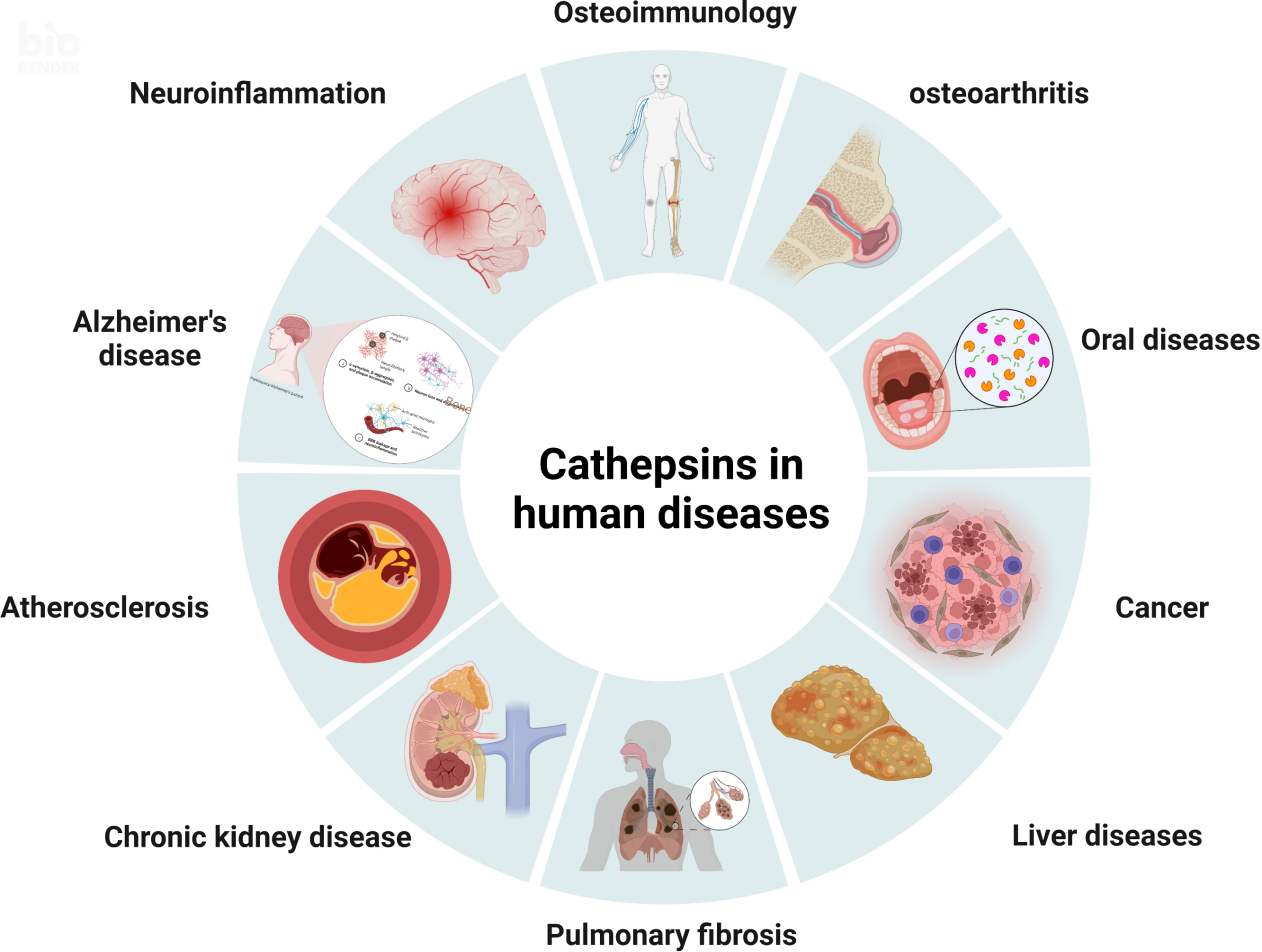
The pathological roles of cathepsins. Various diseases, including osteoimmunology, osteoarthritis, oral diseases, cancer, liver diseases, pulmonary fibrosis, chronic kidney disease, atherosclerosis, Alzheimer’s disease, and neuroinflammation, have been linked to cathepsins.

The word cathepsin, which means “to digest”, initially originated from the Greek kathepsein. It was first used to refer to the idea of proteases that are found in the endosomal/lysosomal proteolytic system and are active in a mildly acidic environment (3). Cathepsins are lysosomal globular proteases, and their classes include serine proteases, cysteine proteases, and aspartyl proteases (4). The eleven cathepsins that make up the cysteine protease family which have been sequenced so far are cathepsins B, H, L, S, C, K, O, F, V, X, and W (5). Various diseases, including atherosclerosis, chronic kidney disease, pulmonary fibrosis, neuroinflammation, osteoarthritis, rheumatoid arthritis, tuberculosis, different cancers, etc., have been linked to cathepsins, as shown in **Figure 1** (6). These cathepsins, however, which differ in their structural makeup and gene sequences, have different mechanisms and functional properties on oral-related diseases. Recent studies have made some latest advancements addressing the connection between cathepsins and oral diseases, which may serve to further elucidate the associated mechanisms of oral diseases and contribute to the development of novel treatment options. For instance, cathepsins may contribute to the degradation of collagen in dental caries, alter oral cancer cell invasion as well as the process of bone resorption in periodontitis and apical periodontitis. They are also more strongly expressed in oral lichen planus.

In this review, we will select dental caries, periodontitis, oral cancer, periapical lesions, and oral lichen planus among the common oral diseases to illustrate the relationship between cathepsins and oral diseases. Starting with the pathophysiology, we’ll go over cathepsins’ structure-function characteristics and regulatory processes in tissue and cells before moving on to potential future therapeutic strategies. By outlining the connection between cathepsins and the development of oral diseases, it is believed that we can more effectively look for treatments, lessening the burden and suffering which oral diseases place on patients.

## 2 Overview of cathepsin characteristics

Cathepsins in the human body are divided into three categories: cysteine ​​proteases, serine proteases, and aspartic proteases. The cathepsins B, C, F, H, K, L, O, S, V, X, and W are the eleven cysteine proteases. Two serine proteases include cathepsins A and G while aspartic proteases contain cathepsins E and D (**Table 1**) (42). The different cathepsin synthetic pathways are nearly identical, however, their structures, catalytic mechanisms, and cleaved proteins are the characteristics that distinguish them (43). The processing and activation of lysosomal proteases are closely related to several features of the intraorganellar environment, including pH within the organelle, processing proteases and membrane proteins, glycosyl side chains of membrane proteins, as well as lipid composition of organelle membranes (44). The specific physiological functions of cathepsins are due in part to the distinctions in their localization inside and outside the cells (45). A slightly acidic environment, such as in lysosomes, is necessary for the lysosomal cathepsins to function at their best (46). Cysteine ​​cathepsins, which are homologous enzymes with a wide range of functions in most tissue and cell types, stand out among the cathepsins. Cysteine cathepsins could take a role in antigen processing and proteolytic enzymes activation thanks to their highly focused and targeted proteolytic action. Additionally, they have a role in extracellular matrix remodeling during tumor cell invasion and wound healing (47–49). Cathepsin B, for instance, which performs as an extracellular matrix protein, could affect cellular processes like angiogenesis as well as metastasis and have an impact on tumor growth, migration, and invasion (50). Besides, for the treatment of chronic inflammatory and autoimmune diseases, cathepsin C appears to be a promising therapeutic target to reduce protease-driven tissue deterioration (51). Moreover, cathepsin K is crucial for the treatment of osteoporosis and is also involved in cell protein turnover, collagen degradation, extracellular matrix remodeling, maintenance of wound healing, tumor growth, and metastasis (52–55).

**Table 1:** Experimental evidence of cathepsins involved in the pathogenesis mechanisms of various disease development.

Categories		Expression in specific cells/tissue	Localization	Pathology	References
cysteine protease	cathepsin B	Neuron, Microglia, Human PBMCs, Type-1 alveolar epithelial cells, Hela cells, Human pancreatic cancer cells, Thyroid carcinoma cells, Liver	Cytosol, Mitochondria, Nucleus, Exosomes and Golgi apparatus	Apoptosis, Inflammation, NLRP3 activation, Oxidative stress, Hepatic steatosis, Ferroptosis, Thyroid malignancies, Osteoarthritis, Alzheimer's disease, Cancer	(7)
	cathepsin C	Neutrophils, Mast cells, Monocytes, Macrophages, Cytotoxic lymphocytes, Natural killer cells, Lung, Spleen, Kidney, Liver	Lysosomes, Golgi	Apoptosis, Pro-inflammatory granule-associated serine proteases activation, NLRP3 activation,Inflammation, Haim-Munk syndrome, Asthma, Rheumatoid arthritis, Multiple sclerosis, COPD, Cystic fibrosis, Sepsis, Cancer	(8–10)
	cathepsin F	Human alveolar macrophage, Senescent human skin cells, Cervical cancer cells, Gastric cancer cells, Heart, Brain, Skeletal muscle, Testis, Prostate, Ovary, Colon	Lysosomes	Apoptosis, Cervical cancer, Gastric cancer	(11, 12)
	cathepsin H	Human prostate cancer cells, Osteosarcoma cells, Pro-monocytic cells, Macrophages, Thyrocytes, Kidney	Lysosomes, Secretory vesicles	BMP4 protein degradation, Protein degradation, Mature neuropeptide substrates degradation, lysosomal degradation of pre-hormones, Pro-granzyme B in cytotoxic lymphocytes processing, Active peptide neurotransmitters production, Angiogenic switching, Tumor vasculature regulating, Colorectal, Pancreatic, Prostate cancer, Glioma, Melanoma, Breast cancer, Fibrous meningioma, Arthritis, Lung cancer	(13–17)
	cathepsin K	Osteoclasts, Epithelial cells, Glioblastoma cells, Brain cells, Neurons, Glial cells, Bronchial cells, Macrophages, Endothelial cells, Multinucleated giant cells, Langhans cells, Fibroblasts, Lymphocytes, Lung, Heart, Renal tumors,	Lysosomes	ECM degradation, Bone metastasis, Collagen degradation, Growth factors degradation, Stem cell Differentiation, Cellular protein turnover, Extracellular matrix remodeling, Wound healing, Autoimmune diseases, Obesity, Lung fibrosis, Osteoporosis, Rheumatoid arthritis, Stroke, Schizophrenia, Cardiac dysfunction, Myocardial infarction, Atherosclerosis, Giant cell tumors, Glioblastoma, Renal tumors, Cerebral aneurism, Chondrosarcoma, Melanoma, Bone cancer, Prostate cancer, Breast cancer, Cancer, Renal cell carcinoma	(18, 19)
	cathepsin L	Macrophages, Microglia, Cancer cells, Tumor-associated fibroblasts	Lysosomes, Nucleus	NLRP3 activation,Apoptosis, Autophagy, Antigen processing and presentation, Spike protein processing, Rheumatoid arthritis, Atherosclerosis, Liver fibrosis, Type I and II diabetes, Cardiac diseases, Bone diseases, Kidney disorders, Immune disorders, Bone turnover, Muscle degeneration, Cancer metastasis	(10, 20–22)
	cathepsin O	Macrophages	Lysosomes	Extracellular matrix degradation, Hormonal regulation, Immune activation, Catabolism of intracellular proteins mediation, Macrophage function activation, Bone resorption activation	(23, 24)
	cathepsin S	Antigen-presenting cells (APCs), B-cells, Dendritic cells, Macrophages, Spleen, Lymphatic system, Cancer	Lysosomes	Inflammation, NLRP3 activation, Pro-inflammatory cytokines and proteases upregulating, Apoptosis, Angiogenesis, Antigen processing and presentation, ECM and BM proteolysis, Decorin degradation, Proteoglycan 4 degradation, PAR2 cleavage, FKN cleavage, Tuberculosis, COPD, Cardiovascular diseases, Atherosclerosis, Cystic fibrosis (CF), Sjögren’s syndrome, Idiopathic pulmonary fibrosis, Lupus, Obesity, Pain, Cancer, Arthritis, Neuropathy	(10, 25)
	cathepsin V	Macrophages, Cornea, Thymus, Heart, Brain, Skin, Testis, Lung bronchus, Kidney, Esophagus, Gastrointestinal tissue	Lysosomes	Antigenic peptides release, Intracellular degradation of elastin, Corneal neovascularization, MHC class II molecules maturation, Elastin fibrils turnover, Intra- and extra-cellular substrates cleavage, Hypertension, Keratoconus corneas, Myasthenia gravis, Atherosclerosis, Aortic aneurysm, Breast cancer, Squamous cell carcinoma, Colorectal cancer	(26)
	cathepsin X	Monocytes, T-lymphocytes, Macrophages, dendritic cells, Neuronal cells, non-hematopoietic bone marrow cells, gastrointestinal tract	Lysosomes	Inflammation, Phagocytosis, maturation, proliferation, migration, adhesion, and the signal transduction of immune cells, Adhesion and maturation of macrophages and dendritic cells, Clathrin-dependent phagocytosis suppression, Hormone signaling regulation, Phagocytosis, Signal transduction, Cancer, Neurodegenerative disorders, Inflammatory diseases	(27–29)
	cathepsin W	NK cells, CTLs	Lysosomes	Chronic inflammatory bowel disease, Autoimmune gastritis	(30, 31)
serine protease	cathepsin A	Fibroblasts, Macrophages, Stimulated cytotoxic cells, Melanocytes/melanoma cells	Lysosomes	Protein degradation, Peptic hormones and biologically active peptides activation/inactivation	(32, 33)
	cathepsin G	Neutrophil leukocytes, B cells, Primary human monocytes, myeloid dendritic cells, plasmacytoid dendritic cells	Exosomes, Lysosomes	Immune reaction, Pathogen clearance, Inflammation, ECM degradation, Platelet activation, Apoptosis, Antigen processing, Blood pressure control, Thrombogenesis inducing, Vascular permeability increasing, Rheumatoid arthritis, Systemic lupus erythematosus, Autoimmunity, Chronic pulmonary diseases, HIV infection, Tumor progression and metastasis, Photo-aged human skin, Papillon–Lefèvre syndrome, Chronic inflammatory pain	(34, 35)
aspartic protease	cathepsins E	Erythrocytes, Lymphocytes, Macrophages, Microglia, dendritic cells, PDAC tissue	Plasma membranes, Endosomal structures, Endoplasmic reticulum, Golgi apparatus, Lysosomes	Protein turnover, Immune response regulation, Apoptosis, Neurodegeneration, Pancreatic duct adenocarcinoma, Breast cancers, Gastric, cervical, esophageal and lung adenocarcinomas, Squamous cell carcinoma, Bladder carcinoma, Ovarian carcinoma	(36–39)
	cathepsins D	All cells, tissue and organs except mature erythrocytes	Lysosomes	Lysosomal digestive process, Apoptosis modulation, Protein degradation, Proteolytic activation of hormones and growth factors, Myocardial infarction, Alzheimer’s disease, Acute kidney injury, Huntington’s disease, Parkinson’s disease, Pancreatitis, coronary disease, Aggressive forms of breast cancer	(38–41)

## 3 Cathepsins in dental caries

### 3.1 Cathepsin B, L and K in the pathogenic mechanism of dental caries

Dental caries is the most common oral disease in the world, characterized by demineralization of tooth hard tissue and an imbalance in the equilibrium between tooth minerals and biofilms. Microbial, behavioral, genetic, and environmental factors may all have some bearing on the occurrence and progression of caries, which is defined as a multifactorial disease (56, 57). The chemico-parasitic theory, which contends that the bacteria in dental plaque biofilms use carbohydrate metabolism to make acid and that the consequent acidic pH can promote enamel demineralization, is frequently used to explain the origin of enamel caries. This theory is also applicable to the occurrence of dentin and root caries, but due to the presence of organic substances such as collagen in dentin and root, the proteolysis theory, which proposes that protein degradation contributes to dentin and root caries, has gained popularity in recent years (58).

**Figure 2:**
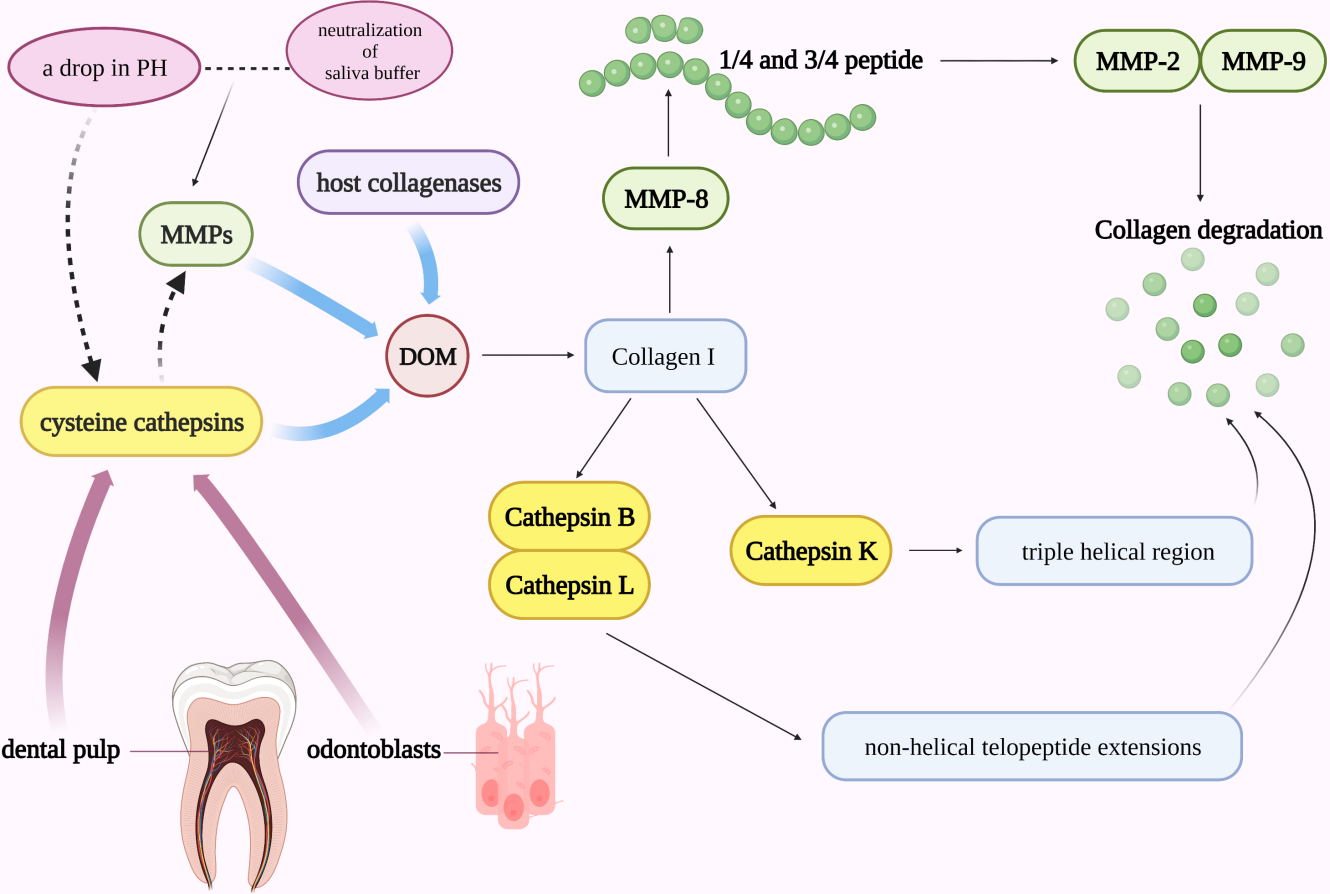
Cathepsins in the pathogenic mechanism of dental caries. DOM may be hydrolyzed by host collagenases, MMPs, and cysteine cathepsins. A drop in pH and the neutralization of saliva buffer can activate the functional activity of MMPs and degrade DOM. Collagen I was initially cut into 1/4 and 3/4 peptide fragments by collagenase MMP-8 and then identified and destroyed by gelatinases MMP-2 and MMP-9. Cathepsin B and L can cleave in the non-helical telopeptide extensions of collagens while cathepsin K can cleave collagen in the triple helical region. MMPs and cysteine cathepsins may work in concert to speed up the development of caries.

The mineral (biological hydroxyapatite) and organic matrix make up about 70% and 20% of the dentin respectively, and the rest of the dentin is water. Collagen makes up the majority of the organic matrix in dentin (around 90% type I collagen and 10% noncollagenous proteins) (59). The organic matrix of dentin is exposed during the demineralization process in dental caries and erosion, with the top layer being a totally demineralized organic matrix (DOM). Protecting DOM is essential for reducing the rate of dentin erosion because DOM can stop ion diffusion and exchange in the demineralizing area (60). Dentin may be remineralized by retaining the collagen fiber scaffold of DOM and supplementing it with the appropriate minerals (61). However, DOM may be hydrolyzed by host collagenases, matrix metalloproteinases (MMPs), and cysteine cathepsins (**Figure 2**) (62). MMPs in human dentin have been identified to include MMP-2, MMP-3, MMP-8, and MMP-9 (63). In the course of dentin erosion, a drop in pH and the neutralization of saliva buffer can activate the functional activity of MMPs and degrade DOM by breaking down collagen. Collagen I was initially cut into 1/4 and 3/4 peptide fragments by collagenase MMP-8, and these peptide fragments with unstable triple helix structures were then identified and destroyed by gelatinases MMP-2 and MMP-9 (**Figure 2**) (59).

Along with MMPs, cysteine cathepsin also plays a significant role in the etiology of dental caries. Cathepsins are involved in the degradation process of collagen: Cathepsin B and L can cleave in the non-helical telopeptide extensions of collagens (64), while cathepsin K, a unique protease that is both a telopeptidase and a collagenase, is the only cysteine cathepsin capable of cleaving collagen in the triple helical region and involves in the absorption of mineralized tissues under normal and pathological conditions (**Figure 2**) (65, 66). It has been established that human dental pulp tissue, odontoblast cells, and intact dentin all express cysteine cathepsins (67). The cysteine cathepsins derived from odontoblasts or dental pulp may be crucial in active caries lesions. Nascimento FD et al. found that in active caries, the activity of cysteine cathepsins in carious dentin rose dramatically with increasing depth (near to dental pulp), particularly in young patients (68). Additionally, MMPs and cysteine cathepsins may also work in concert to speed up the development of caries (**Figure 2**). The first proof that cathepsin K can cleave and activate proMMP-9 in an acidic environment came from the study by Christensen J et al. (69) The synergistic effect of cathepsins and MMPs in the etiology of caries may also be explained by their interaction with pH in collagen degradation. Cathepsins are automatically activated at low pH, while it is mostly unstable and inactive at neutral pH. MMPs, on the other hand, are neutral proteases, but their latent form can be triggered at acidic pH and then neutralized. For example, during dentin invasion, a decrease in pH may activate cathepsin B and then enhance the activity of MMPs by inactivating MMP-specific tissue inhibitors TIMP-1 and TIMP-2 (70). Since MMPs in dentin can be further activated by cathepsins under acidic circumstances and the dentin matrix can be degraded after pH neutralization (71).

### 3.2 Functions of cathepsins in the treatment of dental caries

Given the possible mechanisms of cathepsins in the development of dental caries, novel methods based on the corresponding inhibitors of cathepsins to protect the organic matrix may be beneficial in postponing or halting the progression of dental caries. Santiago AC et al. first showed that a sugarcane cystatin (namely, CaneCPI-5) had a high binding capacity to dental enamel and is highly inhibitory to cathepsin B, K, and L, implicating the ability of this protein to prevent tooth decay by modifying AEP, an acellular film that functions as a diffusion barrier or selective permeation membrane formed on the tooth surface by selective adsorption of salivary proteins and glycoproteins. The new cystatin forms a strong bond with the dental enamel, consequently shielding it from acid erosion (72). According to a study, cathepsin B and K are effectively inhibited by the Ag^+^ in silver diamine fluoride (SDF), which may prevent the advancement of caries by either blocking the active site of collagen to protect it from enzymatic attack or by inactivating enzyme-mediated collagen degradation (66). Besides, molecular docking analysis showed that chlorhexidine (CHX) interacted strongly with the S2~S2 ‘subunits of cysteine cathepsin B, K, and L in a very similar manner, and proved capable to inhibit cathepsins in the dentin pulp complex (73). An increase in the concentration dependence of the elastic modulus of demineralized dentin and surface mineral deposition was seen when CHX-treated dentin was incubated in simulated body fluid, suggesting that cathepsin inhibition may enhance dentin collagen remineralization (74). It is reported that an increase in NaF concentration may have an inhibitory effect on cathepsin K, thereby slowing down the degradation of the dentin matrix, significantly enhancing the longevity of resin-dentin connections as well as preventing dental caries (75). In addition, dentin’s acid resistance may be increased by the application of E-64 (a cysteine cathepsin inhibitor), probably as a result of the efficient protection of DOM. Two mechanisms may be involved: One explanation is that it significantly lowers the level of C-terminal peptide (CTX), a marker of the extent of cysteine cathepsin-induced matrix breakdown. The second possibility is that E-64 not only inhibits the collagen degradation ability of cysteine cathepsin but also blocks the enzymatic activation of MMPs by inhibiting cysteine cathepsins, thereby reducing the activity of MMPs (70).

## 4 Cathepsins in periodontitis

### 4.1 Cathepsin K, B and S in the pathogenic mechanism of periodontitis

Periodontitis is a chronic inflammatory destructive illness that is tightly linked to the interactions between bacterial products, various cell populations, and inflammatory mediators. Periodontitis is brought on by dental plaque, which can build up on the surface of teeth to form a complex and varied microbial biofilm that contains bacteria like lipopolysaccharide of *P. gingivalis* (LPS), peptidoglycan, protease, lipoteichoic acid, and toxins. After being released by bacteria, these virulence factors can be recognized by Toll-like receptors (TLRs) on the surface of host cells, which in turn trigger host cell signaling, activate the host immune system, produce inflammatory mediators such as cytokines and proteolytic enzymes, and induce tissue destruction and bone resorption (76–78). For example, LPS can activate the TLR-4 signaling pathway to increase the level of osteoclast factors, drive the differentiation of osteoclasts in periodontal tissue, and promote the occurrence of periodontitis (79).

TLRs play a key role in the immune response to periodontitis. TLRs, part of pattern recognition receptor (PRR) family, are essential pattern recognition receptors in the innate immune response and are composed of a leucine-rich repeat (LRR) domain that recognizes ligands, a transmembrane domain, and a Toll/interleukin-1 receptor (TIR) (80, 81). As one of type I transmembrane proteins, TLRs can identify two types of ligand, which in turn initiate signaling processes that induce inflammatory cytokine expression. One is pathogen-associated molecular patterns (PAMPs), which are structural motifs that are particularly important to the life cycle of pathogens, and the other is host endogenous damage-associated molecular pattern molecules (DAMPs), such as extracellular matrix components, heat shock proteins, nuclear cytoplasmic proteins (like high mobility group protein 1 (HMGB1)), and nucleic acids released from damaged somatic cells or organelle (82). TLRs can be located in the plasma membrane (TLR1, TLR2, TLR4, TLR5, TLR6, and TLR11) as well as endosomes (TLR3, TLR7, TLR8, and TLR9). TLRs located in the plasma membrane can identify microbial membrane lipids, proteins, and lipoproteins while TLRs positioned in endosomes can detect both microorganisms and self-derived nucleic acids. The MyD88 dependent pathway is the most widely used signaling pathway for almost all TLRs except for TLR3: When TLRs are activated, a protein polymer called “Myddosome” is formed (synthesizing by the intracellular signaling adaptor myeloid differentiation primary response gene 88 (MyD88) and the serine-threonine kinases interleukin 1 receptor kinases IRAK1, IRAK2 and IRAK4), and this complex, in turn, recruits the E3 ubiquitin ligase tumor necrosis factor R-related factor 6 (TRAF6), which subsequently stimulates transforming growth factor beta-activated kinase 1. Furthermore, a series of pro-inflammatory factors (interleukin-1, interleukin-6, interleukin-8, tumor necrosis factor-alpha, etc.) are produced as a result of the nuclear factor-κB (NF-κB) and mitogen-activated protein kinase (MAPK) pathways being activated (83). Several cathepsins are involved in the TLR pathway in the process of periodontal inflammation.

**Figure 3:**
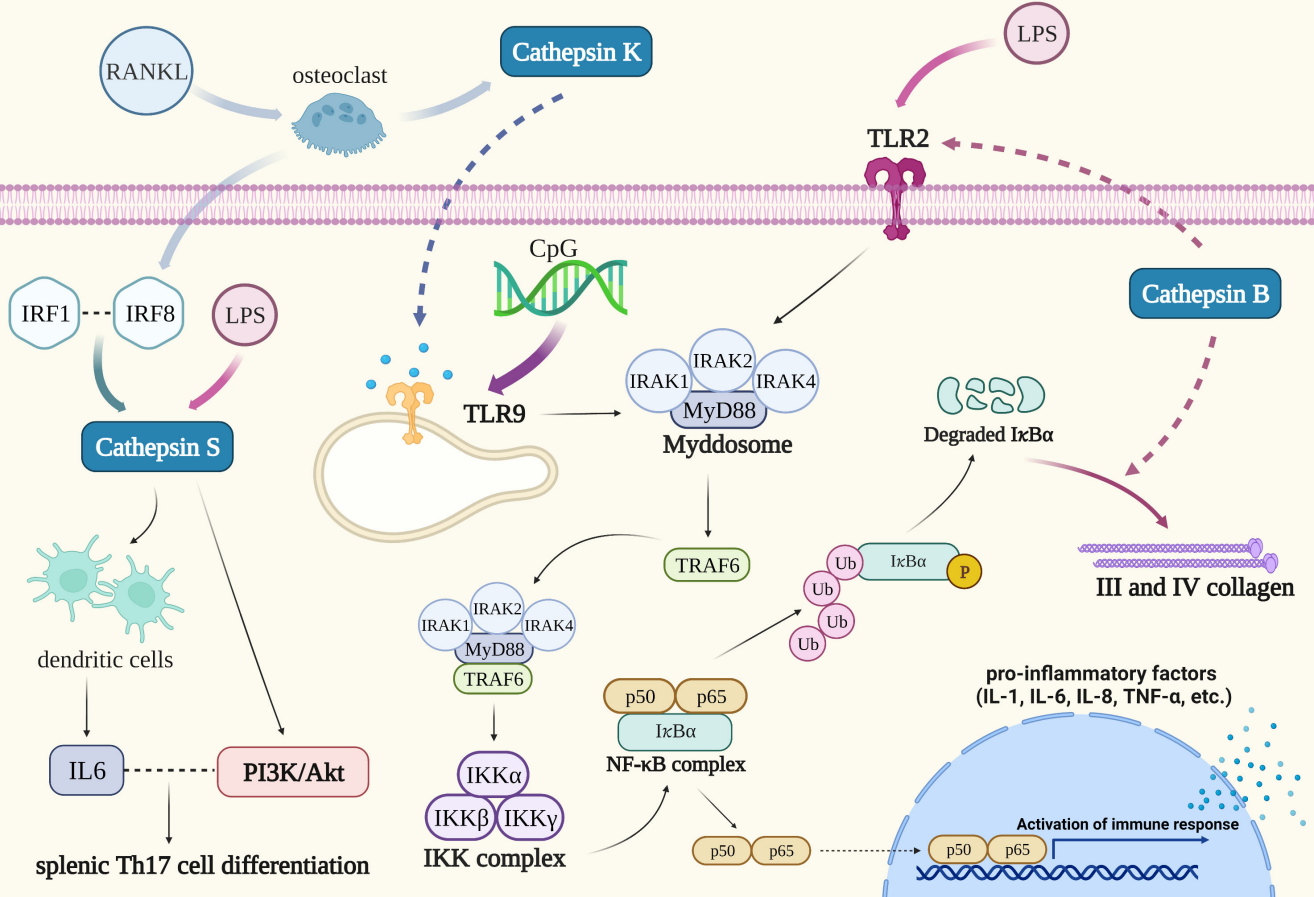
Cathepsins in the pathogenic mechanism of periodontitis. TLR9 is able to identify CpG oligodeoxynucleotides which are present in the bacterial DNA. NF-κB and MAPK are both activated by TLR9 activation, which also triggers the inflammatory response. Blocking cathepsin K could reduce TLR9 expression: The transcriptional levels of MyD88, TRAF6, IRAK1, and IRAK4 in the TLR9 signaling pathway were downregulated. Cathepsin B could reduce the expression of III and IV collagen in condition of chronic inflammation such as periodontitis and oxidative stress through long-term activation of the TLR2/NF-κB signal. Interaction between cathepsin S and IRF8 might involve in periodontitis-associated bone loss. Cathepsin S could stimulate dendritic cells to produce IL-6 when LPS induced an immune response and its activation of PAR2 coupled to the PI3K/Akt signaling pathway could induce IL-6-dependent splenic Th17 cell differentiation.

Patients with periodontitis exhibit varying degrees of periodontal support tissue degeneration, which manifests as alveolar bone resorption and loss of attachment. Cathepsin K is a key modulator of periodontitis-induced bone destruction and may be involved in the immune regulation of periodontitis *via* the TLR9 signaling pathway. TLR9 activation attracts a particular class of adaptors, particularly those dependent on the MyD88 pathway. Nuclear factor-κB kinase (IKK) complex inhibition by TLR9 signaling pathways results in activation of the transcription factor NF-κB and NF-κB signaling cascade. When NF-κB is activated, cytokines are produced, which stimulates the inflammatory response (**Figure 3**) (82). Cathepsin K, a marker of osteoclast activity, is mainly derived from osteoclasts. The formation of osteoclasts from precursor cells and the activation of osteoclasts are both influenced by the receptor activator of the NF-κB ligand (RANKL). According to research by Mogi M et al., periodontitis patients had higher levels of cathepsin K in the gingival crevicular fluid (GCF), and there was a positive correlation between the levels of cathepsin K and RANKL. This suggests that excess of RANKL causes the development of active osteoclasts, which in turn triggers the synthesis of cathepsin K by osteoclasts in periodontal tissue, thereby promoting the bone resorption process (84). When bacterial infection-mediated inflammation occurs, TLR9 is able to identify CpG (a TL9 ligand) oligodeoxynucleotides which are present in the bacterial DNA (**Figure 3**). Pan W et al. found that blocking cathepsin K could reduce TLR9 expression and immune cell infiltration in periodontitis lesions. Cathepsins may be an essential chaperone protein that ensures proper TLRs folding, making them an essential component of the antigen recognition processes. Following cathepsin K suppression, the transcriptional levels of MyD88, TRAF6, IRAK1, and IRAK4 in the TLR9 signaling pathway were downregulated (**Figure 3**). Transcriptional downregulation of the expression of TLR9 pathway-related molecules occurs only after stimulation with CpG, thus revealing the specificity of cathepsin K’s effect on the TLR9 signaling pathway (85).

Cathepsin B can be detected in the GCF of patients with periodontitis. It may be predominantly formed from monocytes that migrate into the gingival crevice, and its level is proportional to the depth of periodontal pockets in periodontitis (86, 87). Recent investigations have suggested that cathepsin B may be implicated in the development of periodontitis. Elkaim R et al. verified that the equilibrium between cathepsin B and its inhibitor cystatin C is upset when *P. gingivalis*, the primary pathogen of periodontitis, infects the epithelium (88). Inferring the progression of the disease from the activity and amount of cathepsin B and cystatin C in GCF may therefore be possible (89, 90). Moreover, the polarization of *P. gingivalis*-infected macrophages toward a pro-inflammatory phenotype requires the activation of NF-κB signaling in the TLR pathway which is also dependent on cathepsin B. The fact that the cathepsin B promoter has an NF-κB binding site suggests that the activation of NF-κB induced by *P. gingivalis* infection and the synthesis of cathepsin B may work in concert (91). Cathepsin B is also engaged in the degradation of collagen in the periodontal tissue of patients with periodontitis. Li X’s study proved that cathepsin B could reduce the expression of III and IV collagen in conditions of chronic inflammation such as periodontitis and oxidative stress through long-term activation of the TLR2/NF-κB signal (**Figure 3**). It has been proven that in fibroblasts, NF-κB binds to IκBα and remains in the inactive cytoplasmic complex as a dimer of p50 and p65 subunits. When fibroblasts were induced with a TLR2 agonist (LPS), NF-κB was activated, then acutely induced phosphorylation, and chronically induced proteolysis degradation of IκBα subunit. Additionally, cathepsin B contributes to the proteolysis and degradation of an IκB subunit to slow down the migration of the NF-κB complex from the nucleus, which may prolong the activation of NF-κB, promote oxidative stress, and lower the expression of III and IV collagen in fibroblasts. The particular inhibitor of cathepsin B may be a successful way to slow the course of periodontitis and repair periodontal tissue, given the involvement of cathepsin B in collagen expression (92).

It is confirmed that cathepsin S can be produced by periodontal ligament cells and its expression is increased during the development of periodontitis, although its functional role in periodontal cells and tissue is unclear. However, it was found that the upregulated expression of cathepsin S generated by interleukin 1β and *F. nucleatum* was nearly eliminated when cells were preincubated with specific inhibitors of MEK1/2 and JNK signaling pathways, indicating that the MAPK pathway may play an important role in mediating the expression of cathepsin S by inflammatory mediators and bacteria. Therefore, it is necessary to do further research to determine whether the control of cathepsin S, an enzyme associated with autophagy, has a significant role in the etiology of periodontitis (93). Song L et al. expatiated that cathepsin S has a binding site for transcription factor interferon regulatory factor 1 (IRF1), and the association of IRF1 with IRF8 may promote the expression of cathepsin S. IRF8 has been demonstrated to be a regulator of osteoclast production in bone metabolism indicating that the interaction between cathepsin S and IRF8 might involve in periodontitis-associated bone loss (94). Dekita M reported that cathepsin S could stimulate dendritic cells to produce IL-6 when LPS induced an immune response and its activation of PAR2 coupled to the PI3K/Akt signaling pathway could induce IL-6-dependent splenic Th17 cell differentiation. As a result, cathepsin S-specific inhibitors may be effective in alleviating periodontitis-associated immunity (95).

In addition, cathepsins are also involved in the mechanisms of other diseases that may interact with periodontitis. The TLR9 signaling pathway and the autophagic response in periodontitis with arthritis have been reported to be sensitive to cathepsin K. The production of the autophagy proteins TFEB and LC3A/B, the secretion of the inflammatory mediators TNF-α and IL-6, the amount of macrophages, and TLR9 are all elevated in periodontitis with arthritis, aggravating bone destruction (96). One of the most important steps in the activation of the TLR9 signaling pathway is the binding of nucleic acids with TLR9 in macrophage endocytic lysosomes (97). According to the findings, TLR9 activation may trigger the cathepsin K-regulated downstream autophagic response. After cathepsin K suppression, levels of TLR9 downstream signaling proteins TRAF6 and IRAK1 as well as autophagy-related proteins decreased, and the number of macrophages was markedly reduced (96). Madapusi Balaji T reported that cathepsin overexpression in periodontitis may enhance the likelihood of developing COVID-19. Cathepsin L levels have been found to be greater in oral mucosa that is pathologically damaged from periodontitis. The cysteine proteases cathepsin B and L have a role in allowing the SARS-corona virus-2 to infect the host cells by mediating the endosomal fusion, which could promote the virus’s strong adherence to host cell membranes (98).

### 4.2 Functions of cathepsins in the treatment of periodontitis

New approaches for treating periodontitis are helped by investigating the potential connection between cathepsins and the pathophysiology of the disease. It is thought that cathepsin K, which is abundantly expressed in osteoclasts and essential for matrix degradation, is an important target enzyme of cysteine peptidase inhibitors that are implicated in bone resorption and may make an ideal pharmacological target to prevent periodontal bone and cartilage destruction (99). In experimental periodontitis models, inhibition of cathepsin K caused an enormous drop in immune cell infiltration as well as a considerable decline in osteoclasts, macrophages, dendritic cells, and T cells (100). Furthermore, due to the function of cathepsin B in the degradation of collagen in periodontal tissue, we could postpone the advancement of periodontitis and repair periodontal tissue using a particular inhibitor of cathepsin B (92). Moreover, pertinent studies have confirmed that cathepsin B consolidated the causative link between LPS and AD, and it’s anticipated that it will serve as a possible therapeutic target for avoiding AD initiation and pathological progression associated with periodontitis (101). Recently, Leguizamon N D P reported a novel phytocystatin (cystatins derived from plants) called CsinCPI-2 with potential anti-inflammatory and osteogenic effects, which could inhibit cathepsin B, cathepsin K, TNF-α, and IL-1β in cultured mouse macrophages. This could become a new therapy for controlling inflammatory diseases such as periodontitis (102). With regard to cathepsin S, its regulation may play a key role in the pathogenesis of periodontitis by affecting the autophagy process and its specific inhibitors may be efficient in reducing periodontitis through related immune pathways (93, 95). In addition, TLRs can bind to bacterial antigens and are related to the innate immune response to periodontal bacteria as well as the activation of adaptive immunity; hence, periodontitis is projected to be treated by medications that specifically target TLRs (76).

## 5 Cathepsins in oral cancer

### 5.1 Cathepsin B, D, L, S and K in the pathogenic mechanism of oral cancer

Cysteine ​​cathepsins as lysosomal peptidases that are critical in triggering cancer progression processes such as tumor proliferation, invasion, angiogenesis, and metastasis through mechanisms including degradation and processing of growth factors, hormones in the extracellular matrix, adhesion proteins, etc. Recent studies have revealed that certain cysteine ​​cathepsins were expressed, abnormally localized, and dysfunctional in cancer stem cells. Cysteine cathepsins may be significant in cancer cells due to the role that cancer stem cells play in tumor initiation, heterogeneity, and treatment resistance (103). Oral cancer is a common high-mortality malignancy, with oral squamous cell carcinoma (OSCC) being the most common and one of the most aggressive types. Despite the use of numerous treatment modalities, including chemotherapy, radiotherapy, and trauma surgery, the mortality rate of oral cancer has not improved considerably in recent years due to the disease’s poor response to chemotherapy and different response to radiation therapy (104). A comprehensive understanding of the molecular mechanism of OSCC is essential for early detection and treatment, thereby improving patient survival (105).

The proliferation, differentiation, apoptosis, angiogenesis, invasion, and metastasis of tumor cells are all regulated by the MAPK signaling pathway (106). Three mechanisms exist for the MAPK signaling pathway to inhibit tumor cell apoptosis. The first mechanism could prevent various stimuli caused by apoptosis. It directly inhibits the activity of the apoptotic end effector caspase-3, followed by caspase-3 inhibition of tubulin hydrolysis, thereby maintaining the integrity of its spindle. The second mechanism may indirectly decrease the activation of caspase-3 through several stimulators, involving cytokines, growth factors, osteopontin, radiation, and B cell lymphoma-2 family proteins (107). The third mechanism may reduce the activity of downstream caspase-3 indirectly by blocking the release of mitochondrial cytochrome C and interfering with the activation of upstream caspase-9 (108). P38 is a sub-pathway of the MAPK signaling pathway and it may be used to drive cell differentiation, apoptosis, and survival. Through the activation of the substrates ATF-2, cAMP response element-binding protein, DNA damage-inducible transcript 3, and myocyte enhancer factor (MEF)2C, the phosphorylated p38 isoforms contribute to the cellular response to environmental stress and inflammation (109, 110).

**Figure 4:**
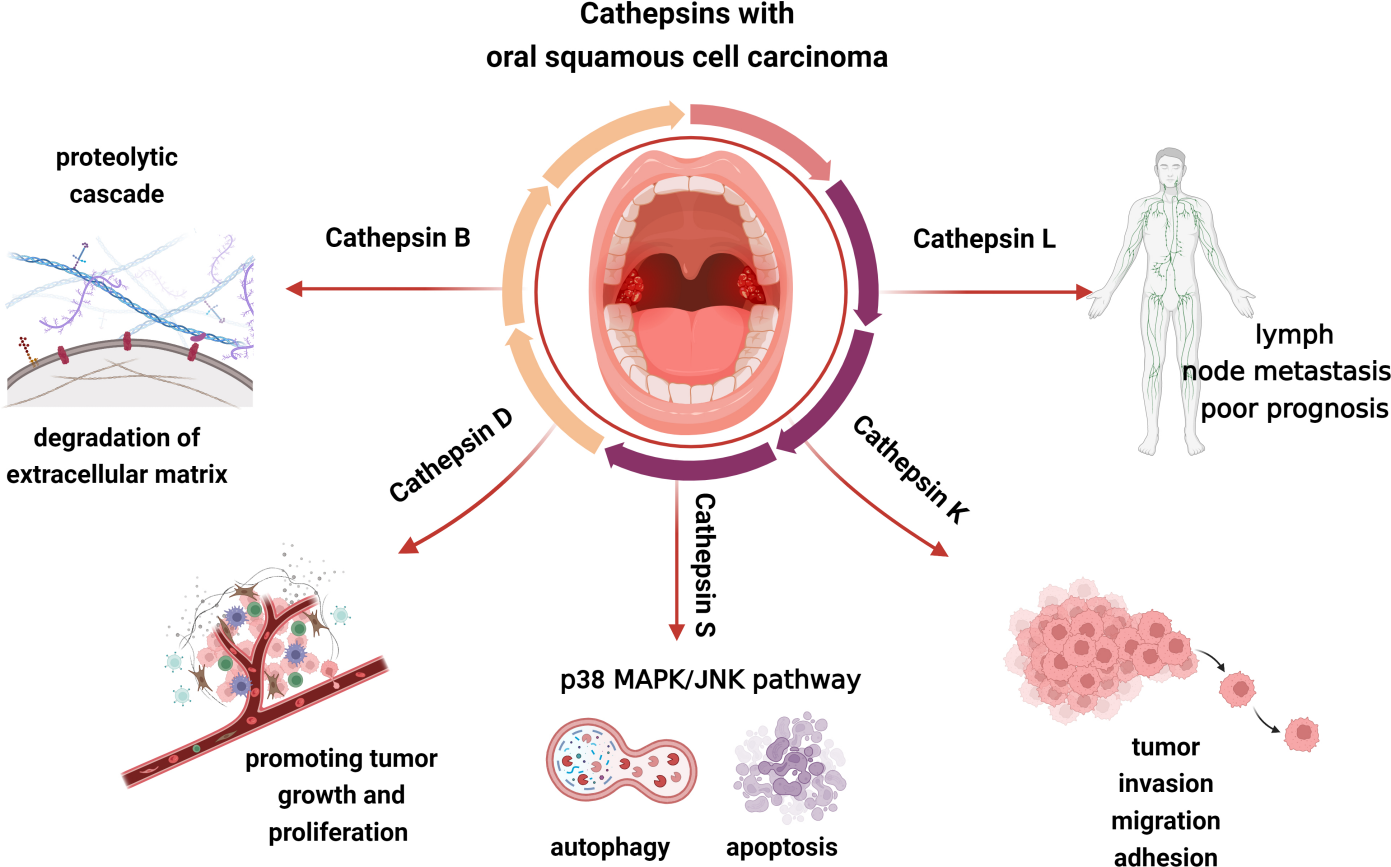
Cathepsin B, L, D, K, and S may be involved in the progress of OSCC, including tumor invasion, migration, adhesion, growth, proliferation, and so on.

**Figure 5:**
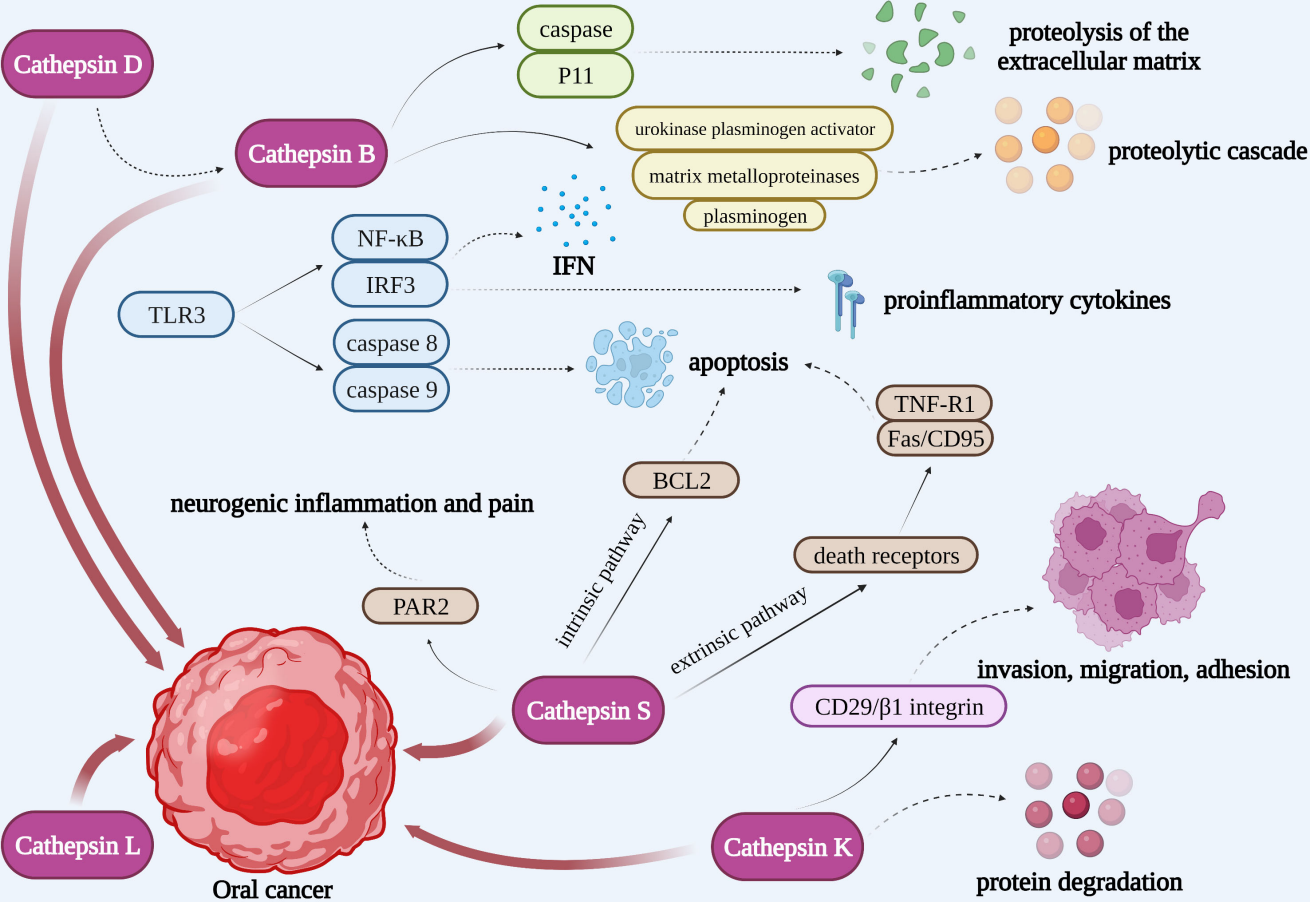
Cathepsins in the pathogenic mechanism of OSCC. Cathepsin B could not only interact with caspase and P11 to activate the proteolysis of the extracellular matrix, but also activate urokinase plasminogen activator, matrix metalloproteinases, and plasminogen to initiate a proteolytic cascade. TLR3 could activate NF-κB and IRF3 to produce IFN and proinflammatory cytokines as well as activate caspase 8 and caspase 9 to induce the apoptosis of tumor cells. Pro-cathepsin B released by tumors can be directly activated by cathepsin D. Cathepsin L may be closely associated with the development of oral cancer. Apoptosis induced by cathepsin S is respectively through an intrinsic pathway mediated by mitochondrial death and an extrinsic pathway mediated by death receptors. Cathepsin S is capable of mediating neurogenic inflammation and pain by cleaving PAR2. Cathepsin K is correlated to OSCC invasion, migration, adhesion and protein degradation.

Tumor cell invasiveness entails cell attachment, proteolysis of the extracellular matrix, and cell migration following mechanism disruption. Since tumor cell-associated proteins can induce cancer cells to escape from the primary site and disrupt the connective tissue barrier of the extracellular matrix as well as the basement membrane, the hydrolytic activity of them affects tumor cell invasion and metastasis (111). Cathepsin B plays an important role in tumor progression, growth, metastasis, and cell invasion. It contributes to the breakdown of basement membrane lysis and extracellular matrix degradation (**Figure 4**). When the extracellular matrix is degrading, cathepsin B could interact with caspase and annexin II tetramer (P11), then localize to key locations where the extracellular matrix is being proteolytically activated. Subsequently, cathepsin B could activate urokinase plasminogen activator, matrix metalloproteinases, and plasminogen to initiate a proteolytic cascade (112). The survival of tumor cells may also be influenced by this mechanism. Type I interferon (IFN) and proinflammatory cytokines such as IL-6 and IL-8 are known to be produced as a result of TLR3 activating the transcription factors NF-κB and IRF3. Moreover, TLR3 can also activate caspase 8 and caspase 9 to induce the apoptosis of tumor cells (**Figure 5**). Cancer cells will undergo apoptosis when cathepsin B inhibits TLR3-mediated apoptosis (113, 114). The involvement of cathepsin B in OSCC has recently been the subject of various investigations. Cathepsin B and Cav-1 gene expression were found to be enhanced in OSCC, as demonstrated by Pakfetrat A et al. (115) The overexpression level of cathepsin B was associated with an increase in the stage and grade of malignancy in OSCC (116). When cathepsin B mRNA was specifically targeted by two ribozymes transfected from 1386Tu oral cancer cells, Wickramasinghe N S et al. observed that the mRNA, protein, and activity levels of cathepsin B were decreased and the motility and invasiveness of oral cancer cells were suppressed. This finding not only proved cathepsin B’s direct contribution to the spread and invasion of oral cancer but also offered a strategy to control the progression and metastasis of oral cancer by targeting cathepsin B with RNA inhibitors (117). When compared to healthy controls, Shabbir A et al. found that OSCC patients had significantly higher levels of salivary cathepsin B. Therefore, cathepsin B can be viewed as a beneficial salivary biomarker with great value in the diagnosis and monitoring of OSCC of different histological grades, which would further enhance the survival rate of OSCC and improve the prognosis (118).

Increased cathepsin D expression is closely associated with tumor metastasis, low histological malignancy, and high proliferative rates (**Figure 4**). Pro-cathepsin B released by tumors can be directly activated by cathepsin D, which then activates the pro-urokinase plasminogen activator (**Figure 5**). Furthermore, the activation of extracellular stores of cathepsin B and D may induce extensive matrix destruction or remodeling through cascades of other enzymes, which might be a major factor in the development of cancer by encouraging the invasion and metastasis of tumor cells (119). Vigneswaran N et al. have identified levels of cathepsin B and D were elevated in OSCC. The severity of biological aggressiveness, propensity to metastasize, histological malignancy, and proliferative activity may be influenced by the greater levels of them (116).

Protein and mRNA overexpression of cathepsin L is frequently detected in oral dysplastic lesions, and their higher levels may be closely associated with the development of oral cancer (120, 121). Nakashima T et al. suggested that the overexpression of cathepsin L was associated with lymph node metastasis and poor prognosis in OSCC and had a potential function to be a prognostic marker (**Figure 4**). The role of cathepsin L in oral cancer progression has not been completely known, though (122). Cathepsin L levels were confirmed to be elevated in malignant cells of squamous cell carcinoma. The local invasion and metastasis of cancer cells to distant sites by catalyzing the degradation of the interstitial matrix and basement membrane may be the mechanism by which cathepsin L could promote tumor cell invasion and metastasis (123). Additionally, in the report on the effect of cigarette smoke condensate (CSC) on oral malignant cells, Nagaraj N S et al. observed a several-fold increase in cathepsin B and L expression and activity in OSCC cells exposed to CSC. That elevation might facilitate OSCC cell invasion. It’s intriguing that specific peptide inhibitors can prevent cells from invading through the Matrigel matrix by inhibiting the activity of the cathepsins. These findings emphasize the critical functions of cathepsins B and L in matrix disintegration and cell invasion in CSC-treated cells as well as the increased risk of metastatic oral cancer in smokers. In the experiments, cathepsin B and L inhibitors could prevent the spread and metastasis of oral cancer, making them a promising potential target for the treatment of smoking-related oral cancer (124).

Da Costa A C et al. conducted that cathepsin S may involve in regulating tumor progression by stimulating apoptosis and autophagy, interfering with angiogenesis, and inhibiting malignant cell migration and invasion (**Figure 4**) (125). Apoptosis induced by cathepsin S is respectively through an intrinsic pathway mediated by mitochondrial death and an extrinsic pathway mediated by death receptors. The intrinsic pathway is regulated by members of the B-cell lymphoma-2 (BCL2) family and its associated death promoter, while the extrinsic pathway is mediated by death receptors on the plasma membrane and then activates tumor necrosis factor receptor 1 (TNF-R1) and Fas/CD95 (**Figure 5**) (126, 127). Autophagy is a stress-inducible process that removes damaged organelles and defective cargos from the cytoplasm, including unfolded proteins. During the beginning and growth of cancer, autophagy contributes to the development of the immunosuppressive microenvironment. It appears to be one of the approaches utilized most frequently in cancer immunotherapy (128). Previously, Zhang L et al. revealed that the phosphatidylinositide 3-kinases, protein kinase B, mammalian target of rapamycin, p70S6 kinase (PI3K, AKT, mTOR, p70S6K), and JNK signaling pathways, which were upstream-mediated by reactive oxygen species (ROS), might play an integral role in the induction of autophagy and apoptosis of mitochondria when cathepsin S was inhibited (129). Besides, Hsieh M J et al. reported that the p38 MAPK/JNK signaling pathway could mediate autophagy and apoptosis in human oral cancer cell lines (130). In addition to carcinogenesis, cathepsin S is capable of mediating neurogenic inflammation and pain by cleaving protease-activated receptor 2 (PAR2) (**Figure 5**) (131). In the oral cancer microenvironment, cathepsin S is both present and active, and when OSCC activates PAR2, it may contribute to oral cancer pain (132).

Cathepsin K is correlated to OSCC invasion, migration, and adhesion in addition to protein degradation (**Figures 4**, **5**). According to research by Yamashita K et al., cathepsin K overexpression could result in the slightly enhanced expression of the adhesion receptor CD29/β1 integrin. However, its precise mechanism for improved invasiveness of OSCC remains unclear (133).

### 5.2 Functions of cathepsins in the treatment of oral cancer

Concentrating on cathepsins may be a novel treatment for OSCC in addition to chemotherapy, radiotherapy, trauma surgery, and other conventional treatments. Cathepsin B has a direct role in promoting the spread and invasion of OSCC. RNA inhibitors could be used to target cathepsin B to control the progression and metastasis of OSCC (117). For gene therapy of OSCC, cathepsin B and cathepsin D may be ideal targets (116). Moreover, Barath Kumar V et al. explored that the plant-derived diarylheptanoid derivative platyphyllenone was a potential anti-metastatic agent, which could reduce the phosphorylation of p38, upregulate the expression of E-cadherin and downregulate the expression of cathepsin L that developing an anti-metastatic effect. The reduction of cathepsin L may affect the epithelial-to-mesenchymal transition and have significant therapeutic implications for highly metastatic OSCC. However, more investigation is needed to figure out the exact mechanism (134). Besides, the inhibition of cathepsin S-induced autophagy in tumor cells could increase the expression of microtubule-associated protein I light chain 3 (LC3) protein, which may then cause the cytoplasmic form (LC3-I) to be cleaved and lapidated into LC3-II. Chen C T et al. elucidated that sulforaphane, a natural phytochemical compound derived from cruciferous plants, could impede the migration of oral cancer cells by suppressing cathepsin S and LC3, suggesting a potential novel approach for the treatment of OSCC (135).

## 6 Cathepsins in periapical lesions

### 6.1 Cathepsin K and C in the pathogenic mechanism of periapical lesions

Periapical lesions are a common pathological condition that could alter the periapical tissue. Microbial invasion and subsequent infection of the root canal system are mainly responsible for the occurrence and development of periapical lesions (136). Periapical lesions mainly comprise radicular cysts, dental granulomas, and abscesses (137). One typical feature of periapical lesions is the destruction of periapical alveolar bone. The enlargement of periapical lesions is associated with the synthesis of proinflammatory cytokines from inflammatory cells. Among them, macrophages are the most prevalent inflammatory cells that cause periapical lesions. The inflammatory process brought on by macrophage infiltration, which may be regulated by NO generation by inducible nitric oxide synthase (iNOS), can hasten the development of apical periodontitis (138). A number of cytokines, including IL-1, TNF, and IL-6, which could activate osteoclast formation and may induce periapical bone destruction, can also be produced by macrophages (139). IL-1α is regarded as a significant stimulator of p

eriapical bone degradation due to its capacity to stimulate the production of NF-κB receptor activator (RANKL), a direct inducer of osteoclasts. IL-6 could mediate helper T cell 17 (Th17) induction to boost the synthesis of IL-17, which is also an important inducer of RANKL (139). The synthesis of these bone resorption cytokines is regulated by cytokines mediated predominantly by T cells. In prior periapical model research, infiltrating macrophages and polymorphonuclear leukocytes in periapical inflammatory lesions showed dramatically higher levels of IL-1a mRNA and protein expression. The synthesis and activity of IL-1α may be related to the regulation of cytokines originating from T cells, particularly those produced by Th1 and Th2 cells. Cytokines produced by Th1 cells are mainly responsible for accelerating the progression of inflammation and can upregulate the expression of IL-1, while cytokines derived from Th2 cells usually have the ability to prevent the onset of inflammation. Th2 cytokines IL-4 and IL-10, for instance, can downregulate IL-1 and Th1-type cytokine production as well as inhibit bone resorption *in vitro* (140). Therefore, the control of apical bone destruction in periapical lesions can be achieved by reducing osteoclast activity or inhibiting osteoclast genesis.

**Figure 6:**
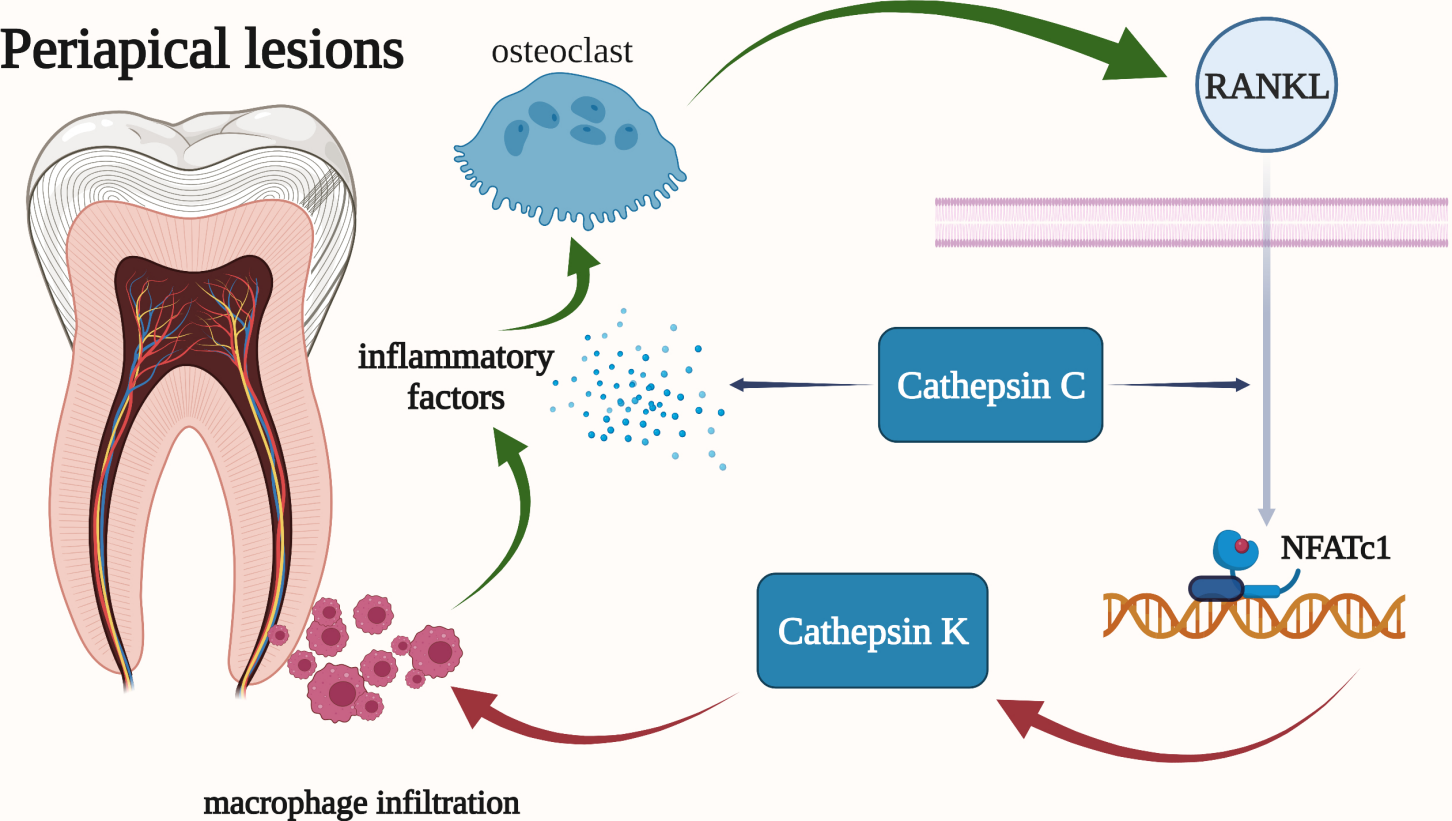
Cathepsins in the pathogenic mechanism of periapical lesions. Cathepsin K is highly expressed in osteoclasts and is regulated by RANKL. And cathepsin K may be involved in the synthesis of pro-inflammatory mediators by activated macrophages. Cathepsin C has been shown to be associated with the overall progression of periapical periodontitis, including bone resorption and the severity of periapical inflammation.

Cathepsin K has long been regarded as a potential target for the treatment of diseases such as osteoporosis. It can degrade collagen type I which is a major component of the organic bone matrix. Osteoclasts express cathepsin K messenger RNA at high levels, and RANKL controls this expression. RANKL firstly stimulates the nuclear factor NFATc1 which could activate T cells and then interact with the cathepsin K promoter to regulate the transcription of cathepsin K. The RANKL pathway can be inhibited by blocking calcium channels to reduce the expression of cathepsin K in osteoclasts (**Figure 6**) (141). Infiltration of inflammatory cells, such as macrophages, is observed in the dilated phase of periapical lesions, and cathepsin K may be involved in the synthesis of pro-inflammatory mediators by activated macrophages. This opinion is corroborated by the *in vitro* detection of cathepsin K RNA expression in RAW264.7 macrophages (139).

It has been demonstrated that cathepsin C refers to the overall progression of periapical periodontitis. Firstly, the elimination of cathepsin C could reduce the severity of periapical inflammation by lowing the local inflammatory factors (TNF-α and IL-1β), chemokines CXCl2, and bone resorption. Besides, the mRNA and protein levels of cathepsin C were significantly increased in acute periapical periodontitis. In addition, macrophages, mast cells, plasma cells, and other inflammatory cells all have high levels of cathepsin C expression in their cytoplasms. Furthermore, the expression of RANKL and the volume of periapical absorption changed more drastically when cathepsin C was activated (**Figure 6**) (142).

### 6.2 Functions of cathepsins in the treatment of periapical lesions

There has been some progress in the cathepsin treatment of periapical periodontitis. Suzuki N et al. demonstrated that the cathepsin K inhibitor NC-2300 significantly down-regulated NO synthesis in macrophages. That indicates cathepsin K inhibitors can effectively reduce the synthesis of pro-inflammatory cytokines and inhibit the progression of periapical lesions (139). Recently, gene therapy for periapical periodontitis has also drawn a lot of interest. Gao B et al. reported that AAV-mediated gene knockout had been successfully applied to the treatment of periapical periodontitis. AAV-mediated RNA knockdown gene therapy silenced cathepsin K *in vivo*, which could in turn inhibit osteoclast function thus significantly reducing pulp disease development, bone destruction, and the severity of inflammatory pulp disease in periapical lesions (143). Besides, Yang W detected that AAV-sh-Ac45 might control periapical inflammation and osteoclast-mediated bone resorption in periapical disorders by inhibiting cellular and extracellular acidification, reducing cathepsin K production, preventing TLRs activation, and interfering with dendritic cell maturation. Moreover, it also indicated that single-target gene therapy had enormous potential in the treatment of periapical periodontitis (144). Cathepsin C knockdown has also been demonstrated to lessen periapical bone resorption and reduce periapical inflammation (142).

## 7 Cathepsins in oral lichen planus

### 7.1 Cathepsin K and B in the pathogenic mechanism of oral lichen planus

A chronic mucocutaneous inflammatory illness called oral lichen planus (OLP) is characterized by the accumulation of inflammatory cells in the lamina propria and the degeneration of the epithelial basal cell layer. Although the exact cause of OLP is unknown, some evidence suggests that antigen-triggered T cells play a role in the disease’s immunological process (145). Both specific and nonspecific mechanisms may contribute to OLP’s pathogenesis. The specific mechanism entails antigen presentation by basal keratinocytes and the killing of antigen-specific keratinocytes by CD8+ cytotoxic T cells. Mast cell degranulation and matrix metalloproteinase activation are two aspects of the nonspecific process in OLP lesions. The combination of these mechanisms may lead to T cell accumulation in the superficial lamina propria, basement membrane disruption, intraepithelial T cell migration, and keratinocyte apoptosis in OLP (146). In the specific mechanism, autologous cytotoxic CD8+ T cells trigger apoptosis of oral epithelial basal cells. Direct activation and indirect activation are the two methods for activating auto-cytotoxic CD8+ T cells. Major histocompatibility complex (MHC)-1-bound antigens on keratinocytes and activated CD4+ lymphocytes are the main drivers of direct activation. Indirect activation results in a rise in Langerhans cells, which is consistent with the upregulation of MHC-II. Following antigen presentation to CD4+ cells, interleukin (IL)-12 stimulates CD4+ T helper cells, which in turn indirectly activates CD8+ T cells through receptor interactions, interferon-gamma (INF-γ), and IL-2 (147).

Siponen M reported that the expression of cathepsin K in OLP was significantly higher than that in normal oral mucosa, indicating that this enzyme may play a role in the pathogenesis of OLP. The cathepsin K-positive cells were found in the superficial lamina propria and the lower epithelia, which may be the most active sites in the pathogenesis of OLP. Additionally, melanocytes in OLP express cathepsin K, which might be a sign of a connected inflammatory condition. Besides, cathepsin K may be involved in the up-regulation of cytokine activity by DC. Interestingly, cathepsin K could coexist with TLR4 and TLR9 in some oral mucosal cells, which reveals that the TLR pathway and cathepsin K may interact in oral lichen planus. Moreover, hyaluronic acid and cathepsin K may involve in the pathological process of OLP, however, further research is needed in this area. Type 1 collagen and other extracellular matrix (ECM) proteins have altered expression in OLP, as well. But whether cathepsin K is involved in the degradation of extracellular collagen and elastin degradation in oral lichen planus remains unclear. Based on the present study, it cannot be confirmed that cathepsin K is active and functional in OLP, in that case, further investigation is required (148).

Georgakopoulou E A et al. suggested that OLP is a tumor-like matrix microenvironment, which is associated with hypoxia, inflammation-related cytokines, transcription factor signals, and decreased blood vessels. The tumor-like microenvironment is also dominated by the protease represented by cathepsin B. Epithelial cells may adopt a state of cell stasis and increase sensitivity to cellular injury as a result of cathepsin B. Additionally, cathepsin B could facilitate cell migration, break down the epithelial integrity, and digest the extracellular matrix (149). In line with this mechanism, researchers have found that cathepsin B expression was increased in oral lichen planus and evident in the cytoplasm and subepithelial matrix of epithelial cells. That might promote the activation of interstitial elements under the epithelium and lead to the formation of the tumor microenvironment (150). Antigen presentation involving MHC-II molecules expressed by Langerhans cells or keratinocytes is one of the particular mechanisms of OLP (151, 152). Cathepsin B is involved in this process. It could cleave the invariant chain to make MHC-II molecules combine with antigens and present them to the immune system (153). Bangsuwan P et al. stated that the expression of cathepsin B in erosive OLP was higher than that in non-erosive OLP, suggesting that the expression of cathepsin B may be related to the severity of OLP. Bangsuwan P et al. also pointed out that the function of cathepsin B may be related to the histopathological characteristics of OLP, such as lymphocyte aggregation, keratinocyte apoptosis, and basal cell degeneration (154). Moreover, Pakfetrat A et al. reported that the gene expression of cathepsin B and caveolin-1 (Cav-1) was elevated in OLP and OSCC. It’s worth noting that the expression of them was higher in OSCC than that in OLP with the increase of lesion stage and grade. Therefore, the detection of these expressions may predict the possibility of malignant transformation of OLP, but additional research is required to identify the relevant markers associated with precancerous lesions (115).

### 7.2 Functions of cathepsins in the treatment of oral lichen planus

According to the above, the pathogenesis of OLP is yet unknown. We hypothesize that the regulation of cathepsin K and B may be beneficial in the treatment of lichen planus, while further research is still needed to determine the exact therapy strategy.

## 8 Conclusions and perspectives

In this review, we explored the relationship between cathepsins and the pathogenesis of dental caries, periodontitis, oral cancer, periapical lesions, and oral lichen planus respectively. Cathepsins are affirmed to be promising as a potential therapeutic target for oral diseases. The specific pathways linking cathepsins to oral diseases, however, has not yet been entirely analyzed, thus further research in this area is required. When it comes to treating oral disorders, cathepsins are still at the hypothetical stage. It is envisaged that in the future, this type of treatment will be made possible in order to avert and lessen the occurrence of oral diseases.

## Author contributions

XL conceptualized, led and funded the project. HJ, XX and ZD conceived the structure of the article and wrote the manuscript. All authors contributed to the article and approved the submitted version.
